# 

**Published:** 1896-05-16

**Authors:** 


					THE HOSPITAL. ABSOLUTELY PURE therefore BEST, Mat 16th, 1896.
CADBURY'S ^ ^ CADBURY'S
COCOA \\. rP COCOA
' The standard of highest purity at present NO ALKALIES USED,
attainable."?Lancet.
No. 503."* Yol. XX.
Saiurday,
May 16th, 1896.
WITH EXTRA NURSING SUPPLEMENT.
raeristered at the General Post Office &t a Newspaper tor Monthly Parts, 6d.;
transmission in the United Kingdom and Abroad.] Ditto per Annnm.8s.6d., po?t tree.

				

